# Machine learning‐based blood pressure estimation using impedance cardiography data

**DOI:** 10.1111/apha.14269

**Published:** 2025-01-13

**Authors:** T. L. Bothe, A. Patzak, O. S. Opatz, V. Heinz, N. Pilz

**Affiliations:** ^1^ Institute of Physiology, Center for Space Medicine and Extreme Environments Berlin Charité—Universitätsmedizin Berlin Berlin Germany; ^2^ Institute of Translational Physiology Charité—Universitätsmedizin Berlin Berlin Germany; ^3^ Department of Cardiology and Angiology Hannover Medical School Hannover Germany

**Keywords:** blood pressure, blood pressure measurement, cardiovascular risk, deep learning, impedance cardiography, machine learning, physiology

## Abstract

**Objective:**

Accurate blood pressure (BP) measurement is crucial for the diagnosis, risk assessment, treatment decision‐making, and monitoring of cardiovascular diseases. Unfortunately, cuff‐based BP measurements suffer from inaccuracies and discomfort. This study is the first to access the feasibility of machine learning‐based BP estimation using impedance cardiography (ICG) data.

**Methods:**

We analyzed ICG data from 71 young and healthy adults. Nine different machine learning algorithms were evaluated for their BP estimation performance against quality controlled, oscillometric (cuff‐based), arterial BP measurements during mental (Trier social stress test), and physical exercise (bike ergometer). Models were optimized to minimize the root mean squared error and their performance was evaluated against accuracy and regression metrics.

**Results:**

The multi‐linear regression model demonstrated the highest measurement accuracy for systolic BP with a mean difference of −0.01 mmHg, a standard deviation (SD) of 10.79 mmHg, a mean absolute error (MAE) of 8.20 mmHg, and a correlation coefficient of *r* = 0.82. In contrast, the support vector regression model achieved the highest accuracy for diastolic BP with a mean difference of 0.15 mmHg, SD = 7.79 mmHg, MEA = 6.05 mmHg, and a correlation coefficient of *r* = 0.51.

**Conclusion:**

The study demonstrates the feasibility of ICG‐based machine learning algorithms for estimating cuff‐based reference BP. However, further research into limiting biases, improving performance, and standardized validation is needed before clinical use.

## 1 INTRODUCTION

Hypertension serves as a crucial risk factor for cardiovascular diseases, such as stroke, heart attacks, and chronic kidney disease.^1^ Accurate and reliable measurement of arterial blood pressure (BP) is of utmost importance for appropriate diagnosis, risk stratification, treatment decision‐making, and therapeutic monitoring.^1^, ^2^, ^3^

In everyday clinical practice, BP measurements are primarily performed using cuff‐based devices.^1^ However, such devices are susceptible to measurement artifacts (disturbed cuff deflation, nocturnal arousals, changes in body posture, or misalignment of patient specific sleep times with the fixed 22‐6 h interval), compromising their accuracy in about 25% of conducted measurements.^4^, ^5^, ^6^ Furthermore, cuff‐based devices exhibit low measurement comfort.^7^

Continuous, cuff‐less, and non‐invasive BP determination has emerged as a potential alternative in the field of hypertension diagnostics.^8^, ^9^ Most of these approaches rely on pulse‐wave‐velocity or analysis of characteristics of the pulse wave.^8^, ^9^, ^10^ Recently, there has been a growing interest in hypertension research in developing algorithms that take various physiological parameters as input for machine learning (including Deep Learning) systems to estimate BP.^8^, ^11^ Especially under circumstances where high‐frequency and reliable BP data is required to ensure patient safety, an alternative to the expensive, labor intensive, and potentially dangerous intraarterial, catheter‐based BP measurement is needed.^12^, ^13^, ^14^ Such situations comprise peri‐operative settings, intermediate and intensive care units as well as situations with currently insufficient BP control such as dialysis.^15^, ^16^, ^17^

Impedance cardiography (ICG) has been identified as a potential approach for BP estimation as changes in BP necessitate and/or induce changes in cardiac function.^18^ ICG is a non‐invasive technique that assesses cardiac function by measuring changes in the thoracic impedance which is directly connected to fluid shifts within the heart and the thoracic vessels. It provides valuable insights into hemodynamic parameters such as stroke volume or cardiac output.^18^

BP dynamics differ in response to mental and physical load.^19^ Acknowledging the need for heterogeneous data, we evaluated different machine learning systems on a dataset containing data collected at rest, during mental and physical load, containing various BP dynamics.

This first‐of‐its‐kind study aims to investigate the possibility of using machine learning algorithms to estimate BP based on ICG and biometrical data.

## 2 RESULTS

### 2.1 Dataset composition

We enrolled 71 young and healthy adults in this study (Table 1).^19^

**TABLE 1:** Dataset composition.

	Total (*N* = 71)		Male (*N* = 34)		Female (*N* = 37)	
	Mean	SD	Mean	SD	Mean	SD
Age in years	21.9	2.8	21.4	2.3	22.3	3.2
Weight in kg	68.8	13.0	79.2	10.1	59.2	6.2
Height in cm	175.2	9.9	183.3	7.1	167.6	4.9

Two participants only completed part two of the experiment (physical load), and one had to stop due to feeling unwell after the Trier social stress test (TSST). A technical issue with ICG (ICG electrodes lost contact) prevented data analysis for all parts of the experiment in one case. As a result, there were valid results for 68 participants for phase one (TSST) and 69 for phase two (ergometer).

### 2.2 Model estimation performance

#### 2.2.1 Systolic BP

For systolic BP values, regression models retrieved the best results, with an unregularized linear regression model showing the best overall model performance. All tested models showed a mean difference of below 1 mmHg (Table 2).

**TABLE 2:** Systolic model performance.

	Diff.	SD	*r*	MAE	RMSE
Linear regression	−0.01	10.79	0.82	8.20	10.79
Lasso regression	0.16	11.01	0.81	8.35	11.01
Elastic net	0.15	11.33	0.82	8.71	11.34
Regression tree	−0.16	13.19	0.72	10.28	13.19
Forest regressor	−0.18	12.11	0.77	9.38	12.12
k‐NN regressor	−0.13	11.29	0.80	8.58	11.29
Support vector regressor	−0.54	11.29	0.81	8.60	11.30
AdaBoost regressor	0.88	12.56	0.75	9.83	12.59
Multi‐layer perceptron	−0.26	11.86	0.78	9.10	11.87

Abbreviations: Diff., mean difference; MAE, mean absolute error; *r*, correlation coefficient; RMSE, root mean squared error; SD, standard deviation.

**FIGURE 1:**
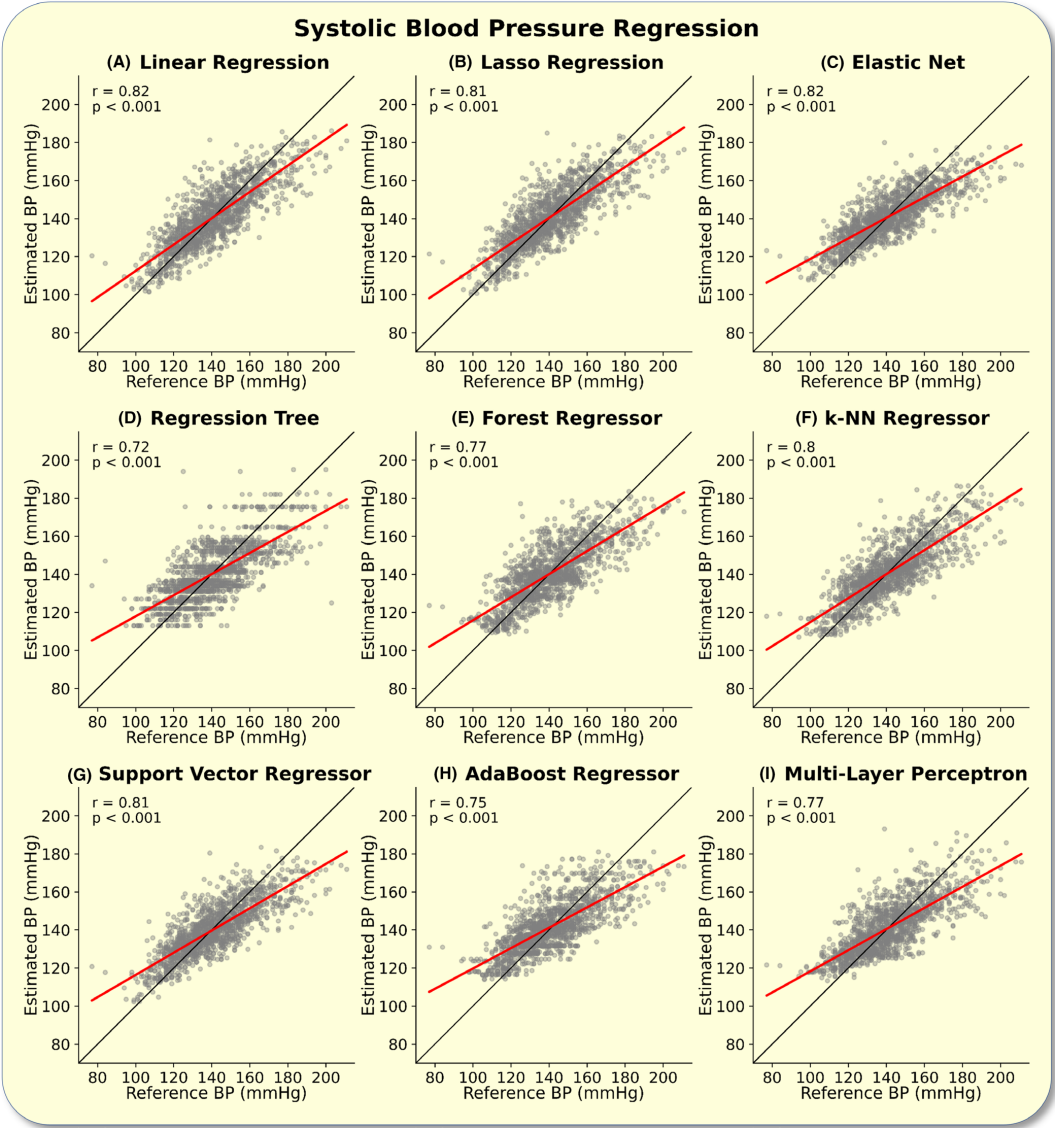
Systolic blood pressure (BP) regression: The figure depicts the regression analysis between the estimated and reference systolic BP. Correlation coefficients and *p*‐values are shown in the upper left corner of each plot. The plots show the regression analysis for the linear regression (A), lasso regression (B), elastic net (C), regression tree (D), forest regressor (E), k‐nearest‐neighbor (k‐NN) regressor (F), support vector regressor, AdaBoost regressor (H), and multi‐layer perceptron (I). The line of identity is plotted in black.

Further, the systolic BP estimations of all tested models correlated with the reference BP values in the regression analysis (*p* < 0.001). All models scored a correlation coefficient of *r* > 0.7. The best performing model (lowest root mean squared error [RMSE]) was the linear regression model (Figure 1).

#### 2.2.2 Diastolic BP

For diastolic BP, the support vector regressor retrieved the best overall model performance. All tested models showed a mean difference of below 1 mmHg (Table 3).

**TABLE 3:** Diastolic model performance.

	Diff.	SD	*r*	MAE	RMSE
Linear regression	−0.02	7.96	0.48	6.19	7.96
Lasso regression	0.07	7.96	0.48	6.19	7.96
Elastic net	−0.02	8.04	0.47	6.26	8.04
Regression tree	0.09	8.91	0.34	7.02	8.91
Forest regressor	−0.08	8.25	0.42	6.51	8.25
k‐NN regressor	−0.36	8.20	0.43	6.44	8.21
Support vector regressor	0.15	7.79	0.51	6.05	7.79
AdaBoost regressor	−0.72	8.71	0.35	6.84	8.74
Multi‐layer perceptron	−0.08	8.43	0.37	6.62	8.43

Abbreviations: Diff., mean difference; MAE, mean absolute error; *r*, correlation coefficient; RMSE, root mean squared error; SD, standard deviation.

**FIGURE 2:**
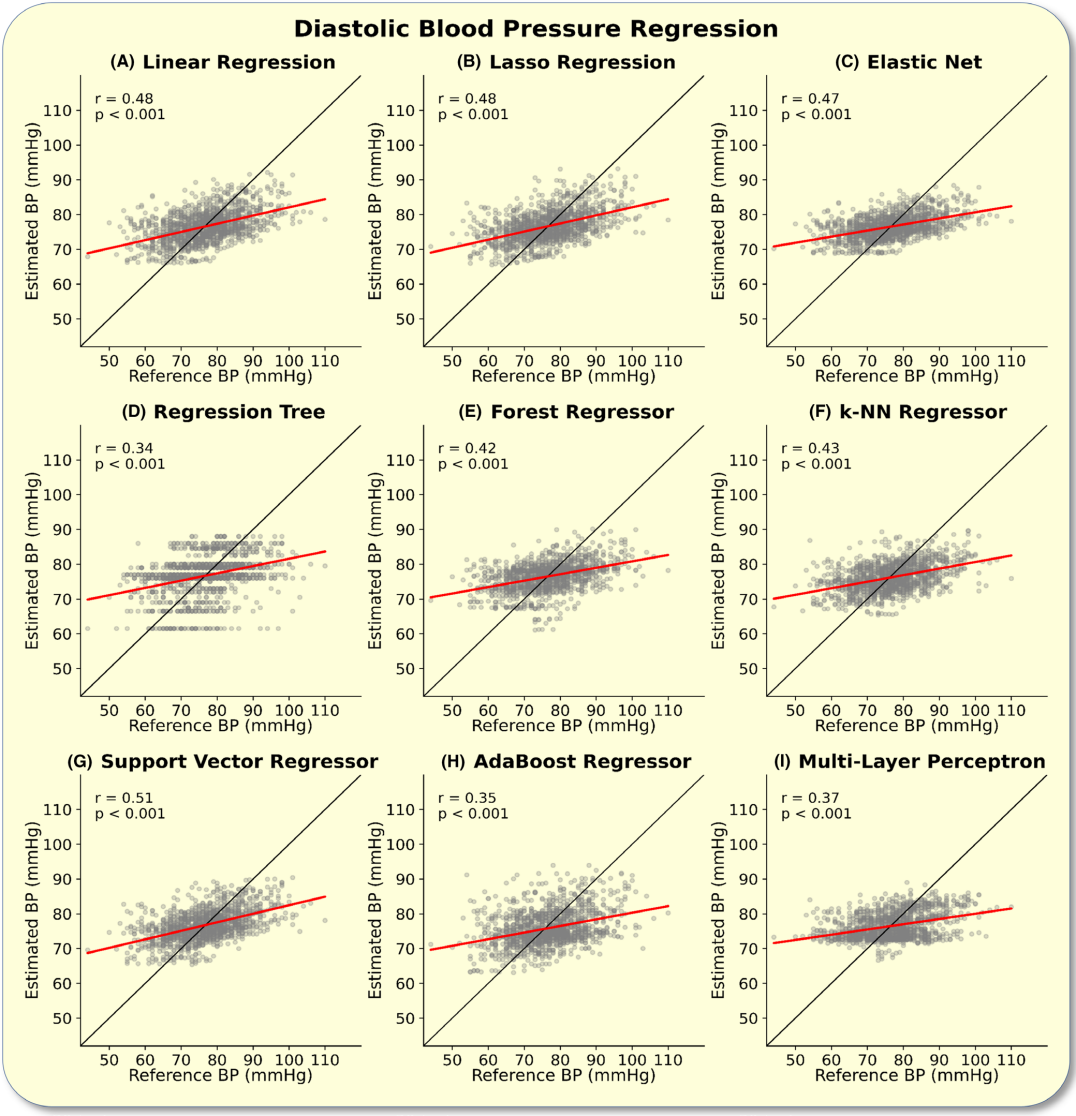
Diastolic blood pressure (BP) regression: The figure depicts the regression analysis between the estimated and reference diastolic BP values. Correlation coefficients and *p*‐values are shown in the upper left corner of each plot. The plots show the regression analysis for the linear regression (A), lasso regression (B), elastic net (C), regression tree (D), forest regressor (E), k‐nearest‐neighbor (k‐NN) regressor (F), support vector regressor, AdaBoost regressor (H), and multi‐layer perceptron (I). The line of identity is plotted in black.

As for the systolic values, the diastolic BP estimations of all models correlated with the reference BP values in the regression analysis (*p* < 0.001, Bonferroni corrected). All models scored a correlation coefficient of *r* > 0.3. The best performing model (lowest RMSE) was the support vector regressor (Figure 2).

#### 2.2.3 Best performing algorithms

**FIGURE 3:**
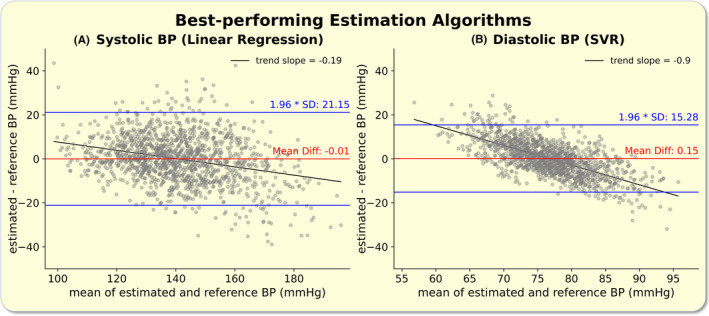
Best performing estimation algorithms: The figure depicts a Bland–Altman analysis for the best performing systolic (A, Linear regression) and diastolic (B, SVR, support vector regressor) blood pressure (BP) estimation algorithms. The mean difference (Mean Diff, red), the limits of agreement (1.96 × SD, blue), and observed bias (trend line, black) are displayed. SD, standard deviation.

Inserting the results from the best performing models (lowest RMSE) for the systolic (linear regression) and diastolic (support vector regressor) BP estimation into a Bland–Altman analysis allowed for intuitive visual data interpretation (Figure 3). Both models showed low coefficients of variation of 0.077 (systolic) and 0.102 (diastolic).

Assessing the data split into data recorded during mental load (TSST) and during physical load (bike ergometer) allowed us to describe the estimation performance more accurately under each of these measurement circumstances.

**FIGURE 4:**
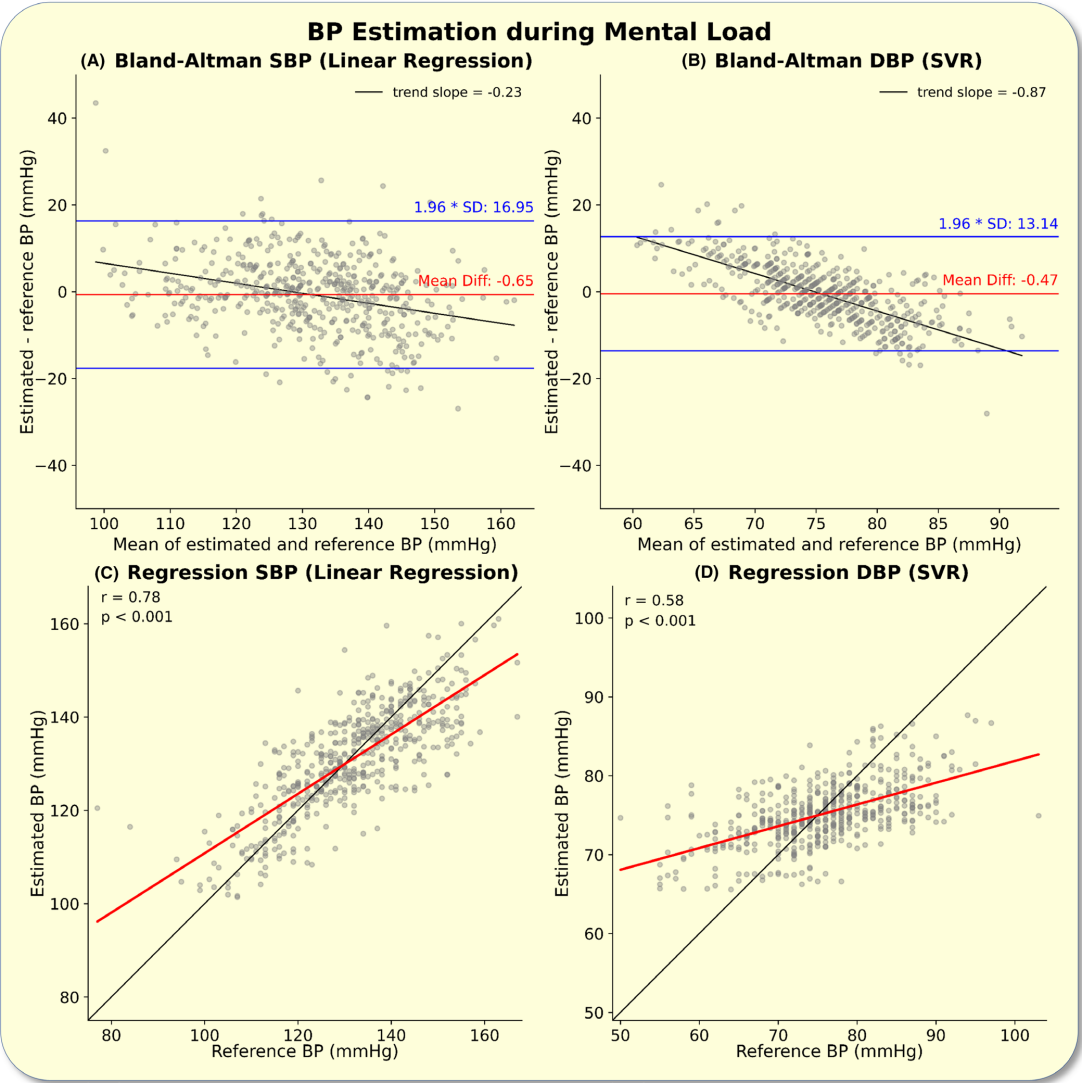
Blood pressure (BP) estimation during mental load: The figure shows the performance metrics of the proposed BP estimation models during mental load. The upper panels depict Bland–Altman analyses for the systolic (A) and diastolic (B) BP estimation. The lower panels illustrate correlation analyses between the reference (cuff) and estimated systolic (C) and diastolic (D) BP. The line of identity is plotted in black. DBP, diastolic blood pressure; Mean Diff., mean difference; SBP, systolic blood pressure; SD, standard deviation; SVR, support vector regressor.

**FIGURE 5:**
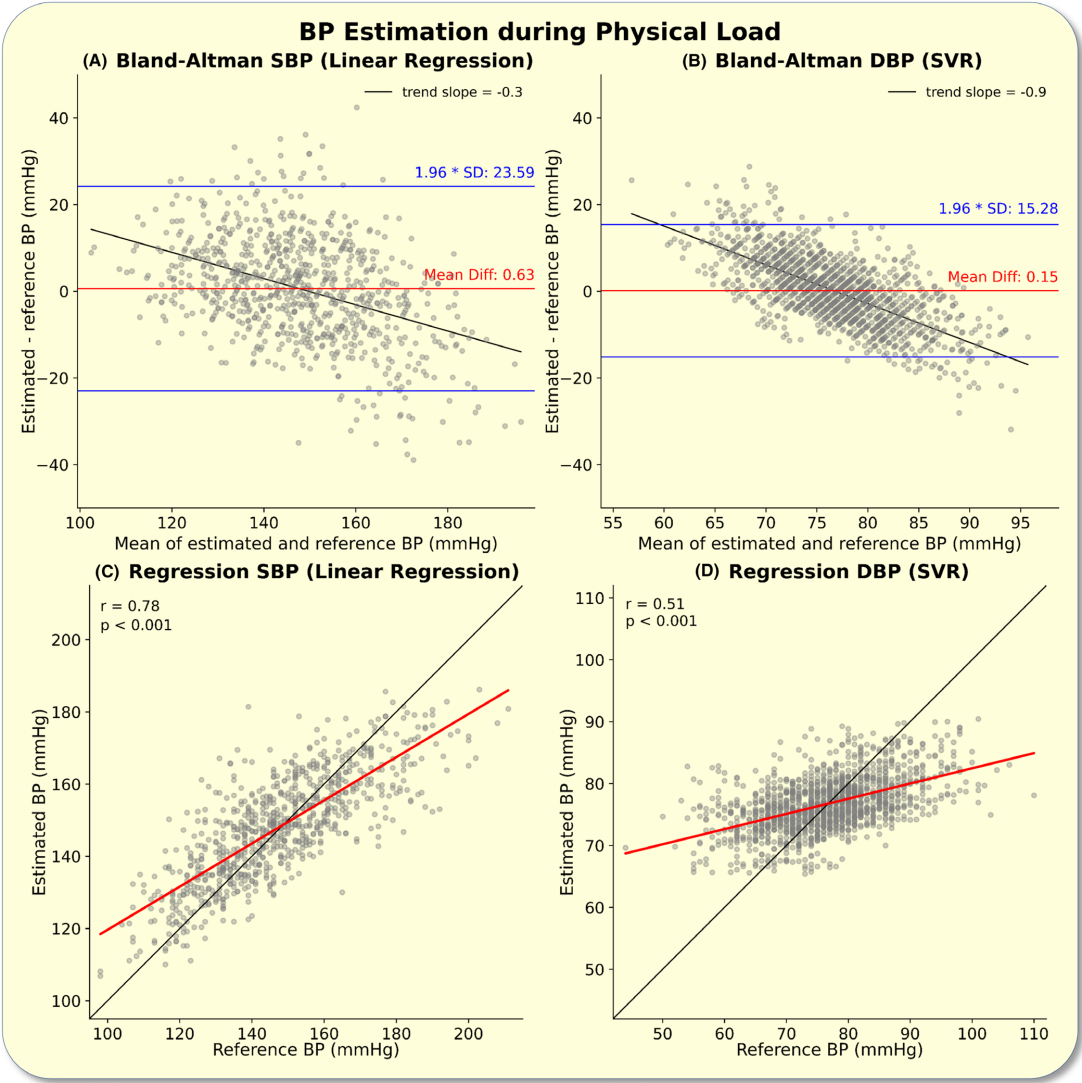
Blood pressure (BP) estimation during physical load: The figure shows the performance metrics of the proposed BP estimation models during physical load. The upper panels depict Bland–Altman analyses for the systolic (A) and diastolic (B) BP estimation. The lower panels illustrate correlation analyses between the reference (cuff) and estimated systolic (C) and diastolic (D) BP. The line of identity is plotted in black. DBP, diastolic blood pressure; Mean Diff., mean difference; SBP, systolic blood pressure; SD, standard deviation; SVR, support vector regressor.

The proposed best performing models for systolic and diastolic BP estimation showed comparable measurement accuracy and correlation measures during mental as well as during physical load (Figures 4 and 5).

The observed biases in all six Bland–Altman analyses were statistically significant (*p* < 0.001) with weaker biases for systolic BP estimations (Figures 3, 4, 5).

**FIGURE 6:**
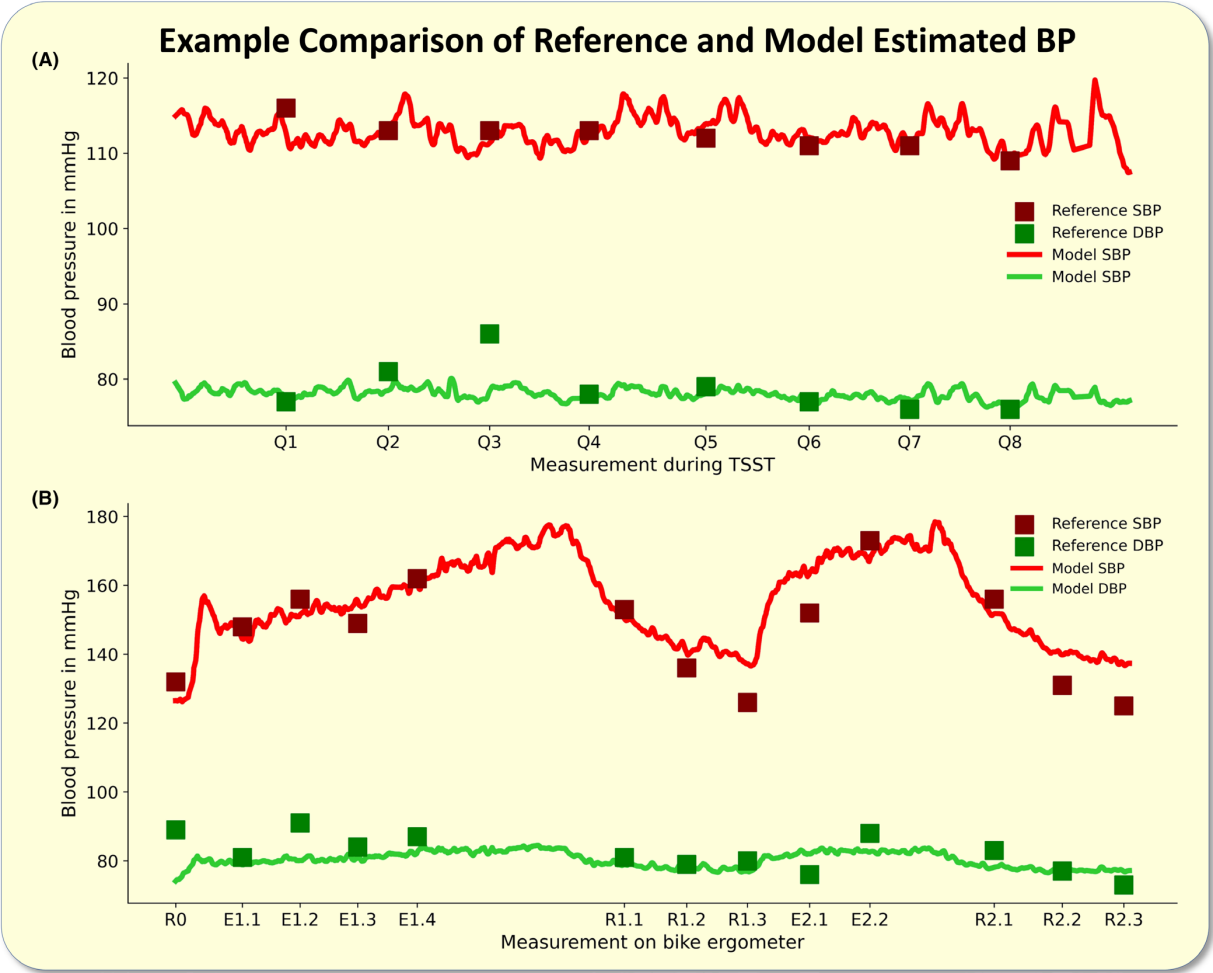
Example comparison of reference and model estimated blood pressure (BP): The figure illustrates the reference (oscillometric) and estimated BP for selected example profiles. The upper panel (A) shows the mental load phase while the lower panel (B) depicts the physical load. The estimated systolic BP was derived via the linear regression model and the diastolic estimation was made from the support vector regressor model. The missing reference values during physical load are due to the exclusion of impaired oscillometric pressure curves. DBP, diastolic blood pressure; E, ergometer load; Q, TSST question; R, rest; SBP, systolic blood pressure; TSST, Trier social stress test.

Plotting the estimated BP curve based on the continuously recorded ICG data from one subject (who's data has not been shown to the model during training) allowed us to demonstrate the model's capability of showing continuous BP estimates. This analysis revealed the close agreement and physiologically expectable BP dynamic during mental and physical load. A rolling average over 10 beat‐to‐beat ICG‐based estimations was used to plot the data (Figure 6).

The best performing models showed no significant difference in mean BP for subject specific mean values during the TSST, both for systolic (0.8 mmHg, *p* = 0.39) and diastolic (0.6 mmHg, *p* = 0.62) measurements. The derived hypertension classification agreed in 70.6% of subjects for systolic and 91.2% for diastolic measurements. For rest measurements, there was a difference of 2.9 mmHg (*p* < 0.01) for systolic and 2.5 mmHg (*p* = 0.03) for diastolic measurements with classification agreements of 57.4% and 86.8%, respectively. Detailed information is presented in Supporting Information S4

### 2.3 *B*‐score analysis

Calculating the *B*‐score revealed the algorithms' predictive advantage above the *B*‐score's *base performances*. The systolic *base performances* were 19.04 mmHg (B1), 26.55 mmHg (B2) and 12.59 mmHg (M), while calculation of the diastolic *base performances* retrieved values of 9.13 mmHg (B1), 12.23 mmHg (B2) and 7.79 mmHg (M).

For the systolic BP estimation, the linear regression model scored a RMSE of 10.79 mmHg which outperformed all *base performance* RMSE values. This led to a defined *B*‐score of 0.386.

For the diastolic BP estimation, the support vector regressor scored a RMSE of 7.79 mmHg which outperformed all *base performance* measures but only slightly beat the standardized deep learning model (M) the *B*‐score provides, leading to a defined *B*‐score of 0.093 (Supporting Information S5).

## 3 DISCUSSION

The results of this first‐of‐its‐kind investigation show the feasibility of machine learning approaches in the determination of arterial BP based on ICG data. Despite the challenging conditions posed by mental and physical load, both systolic and diastolic BP estimation showed small mean differences and a strong correlation with the reference BP. The linear regression model achieved the best performance for systolic BP while the support vector regressor model performed best in diastolic BP estimation. The *B*‐score analysis further indicates that the observed measurement accuracy may be comparable to current cuff‐based devices used for ambulatory BP monitoring.^5^, ^26^ Beyond, the two best performing models provided comparable measurement accuracy during both mental and physical load. These findings suggest that the proposed models may generalize properly across different measurement conditions. However, additional studies are required to confirm this adaptability.

All analyses showed biases (trend slopes) in the BP estimation Bland–Altman analyses. These biases were more pronounced for diastolic than for systolic BP We suspect that these biases are product of models optimizing for a minimal RMSE, the insufficient training data we were able to provide within this study and further exaggerated by the fact that there was low variability within the observed diastolic BP values. The digitality (discrete values) of the predictions made by machine learning algorithms (e.g., Regression Tree) substantiates the intuition of lacking training data being a central limiting factor. It is crucial that these biases are reduced in future research, likely through the availability of more training data and therefore by enabling more complex models. We hypothesize that deep learning models will outperform classic machine learning models and reduce biases once more data is available. Importantly, while this study showed the feasibility of ICG‐based BP estimation, clinical validation in accordance with standardized protocols and further development to mitigate its limitations are needed before this technology can be implemented in clinical practice.^1^, ^20^ At this time, it can neither be advised nor recommended to use an ICG‐based BP estimation model for diagnosing or monitoring hypertension.

The clinical relevance of these findings could be meaningful, though further investigation is needed to fully understand their potential impact. A reliable approach for the precise, non‐invasive, and continuous measurement of arterial BP is dearly needed in various medical scenarios. Acute changes in BP directly indicate situational and possibly life‐endangering changes in perfusion, such as bleeding or shock.^1^, ^32^ Mean BP levels are the most important clinical parameter for overall cardiovascular risk.^1^, ^32^ However, detecting and correctly quantifying dynamic changes in BP is essential in peri‐operative settings and in critical care medicine.^33^, ^34^, ^35^ Only reliable, precise, and ideally continuous measurement can enable these real‐time applications. If validated in accordance to agreed international protocols and shown to be valuable in larger clinical studies, our proposed technology might have the potential to develop into a clinical tool offering a more comfortable alternative to conventional cuff‐based methods while providing more BP values. Peri‐operative monitoring and intermediate care units would greatly benefit from a method which could be easily applied, scaled and which derives reliable and real‐time, beat‐to‐beat BP measurements without the need for invasive and therefore potentially dangerous BP measurement.^8^, ^36^, ^37^, ^38^

Several limitations need to be acknowledged when interpreting the study's outcomes. Our study's focused on a young, healthy, and predominantly non‐hypertensive population. Future research should incorporate more diverse and hypertensive patients to better assess the model's accuracy in clinical populations. Additionally, the study was conducted in a controlled and stationary setting, which may not fully represent the complexities of real‐world clinical scenarios such as ICU‐wards or dialysis centers.^15^, ^16^, ^17^ We acknowledge that using cuff‐based, oscillometric BP measurement instead of the manual auscultatory BP reference method in this study represents a significant limitation. Oscillometric BP measurements are known to have certain limitations, especially when movement artifacts affect the inflation and deflation process.^39^ To address these issues as much as possible, participants were instructed to keep their arms still during measurements. Additionally, we recorded the pressure curves and excluded any BP measurements showing a pressure increase of over 8 mmHg during deflation, following recommendations in the literature, to reduce potential inaccuracies in the data. Nonetheless, this limitation must be considered when interpreting our results.^4^, ^39^ A cuff calibration is needed to start the measurement. This is common within the field of continuous BP monitors (especially amongst the most promising approaches).^8^, ^9^ ICG is susceptible to movement artifacts and transitioning this approach into clinical practice will pose technical challenges. Potential future applications for an ICG‐based BP estimation system could lie in stationary settings without much patient movement, such as post‐surgical surveillance or in the intensive care unit. We do not consider this technology suitable for scenarios involving increased patient movement, such as 24‐h BP monitoring. Lastly, the ICG's measurement site (thorax) and the reference BP measurement site (upper arm) may pose an inherent limitation. While it may be reasonable to assume that thoracic ICG measurements could estimate central aortic (thoracic) BP comparably to brachial BP if trained on respective data, further investigation is needed to confirm this. Given that central aortic BP has been associated with a higher predictive value for adverse cardiovascular events, this may represent a potential area for future research.^40^

## 4 METHODS

### 4.1 Dataset

This study is a secondary analysis of data presented in a formerly published article, which investigated the impact of the pre‐ejection period on pulse‐wave‐velocity based BP estimation.^19^ We included young adults (18–35 years) without known pre‐existing medical conditions or ongoing medication. Exclusion criteria encompassed pre‐existing cardiovascular disease, vasoactive medication, or any injuries that prevented participation in the physical exercise component of the measurement.^19^

### 4.2 Devices

#### 4.2.1 Impedance cardiography

ICG measurements were performed using the *CardioScreen® 1000* (medis Medizinische Messtechnik GmbH, Ilmenau, Germany). This device has undergone validation as bedside monitor named niccomo™.^21^, ^22^ The ICG measurement principle is described in more detail in Supporting Information S1.

#### 4.2.2 BP monitor

We conducted BP measurements using an *OnTrak 90 227* (Spacelabs® Healthcare, Snoqualmie, USA) cuff‐based device.^23^ The pressure of the cuff was continuously recorded via a Y‐connection attached to a pressure transducer. The cuff pressure time series were recorded using a *SOMNOtouch™ NIBP* (SOMNOmedics GmbH, Randersacker, Germany). This enabled us to evaluate the validity of every single BP measurement. As done in the pre‐ejection period focused analysis of the dataset, we excluded measurements with heavily disturbed and therefore ineligible pressure curves. Additionally, we instructed the participants to keep their arms still during measurement.^19^

All BP measurements with an increase in cuff pressure of more than 8 mmHg during cuff deflation (negatively affecting the detection of pulse pressures in the oscillometric signal and therefore greatly affecting measurement accuracy) were excluded, as recommended in recent scientific literature.^4^, ^5^

#### 4.2.3 Ergometer

In this study, we employed a recumbent bike ergometer, the *ergometrics 900 L* (ergoline, Bitz, Germany), with an inclination of 60°. By doing so, we were able to substantiate a well‐controlled physical load regimen, while minimizing upper body movement, ensuring minimal disruptions to both ICG and BP measurements.^19^

### 4.3 Procedure

Seventy‐one young and healthy subjects underwent simultaneous ICG and cuff‐based BP measurement at rest, as well as under mental and physical load.

#### 4.3.1 Resting period

After attaching the devices, the subjects were seated behind a desk. We instructed the subjects to relax and remain at total rest for 2 min. Afterwards, the first BP measurement (rest) was performed.

#### 4.3.2 Mental load

To induce mental stress, we conducted an adapted version of the TSST.^24^, ^25^ The test consisted of a total of eight questions: four questions of a simulated job application interview and four mental arithmetic tasks. The subjects had to answer each question within 1 min. Following the subject's response to each question, we performed a BP measurement while the subjects refrained from speaking.^19^

After the completion of the TSST, we performed a comprehensive debriefing to ensure that the subjects had a consistently positive experience during this part of the experiment.

#### 4.3.3 Physical load

We moved the subjects onto the bike ergometer and started with the physical load protocol as soon as they reached their resting heart.

We instructed the subjects to pedal continuously at a rate of 60 rpm. The resistance load was increased in load levels which were weight‐adjusted and calculated as 0.4 W × body mass in kg.

After 1 min of treading on a load level, we initiated a BP measurement during which the subjects continued to. After completing the BP measurement, we increased the load level as described above. Once the subjects reached 80% of their calculated maximum heart rate (220—age in years), they were instructed to stop pedaling after the subsequent BP measurement.

Subsequently, three BP measurements at recovery were conducted, each starting 1 min after the previous BP measurement had finished.

Thereafter, the subjects performed one load le at the second highest level of the first physical load and then two rounds on the maximum load level.

**FIGURE 7:**
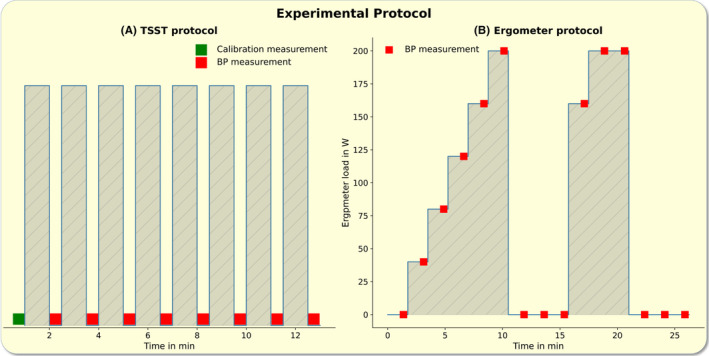
Experimental protocol: The left panel (A) depicts the Trier social stress test (TSST) protocol, with time during answering questions (bars) and automated blood pressure (BP) measurements during the explanation of the next task (red squares). The subjects did not speak during BP measurements. The right panel (B) shows the ergometer protocol, with an increasing physical load, shown for a subject with body mass of 100 kg and five load levels. The green square shows the time of the calibration measurement. BP measurements are indicated by the red squares.

Eventually, three resting BP measurements during the second recovery period were conducted, according to the described procedure (Figure 7).^19^

### 4.4 Model creation and statistical analysis

#### 4.4.1 Model selection

We tested nine different machine learning approaches to evaluate the predictive capability of an ICG‐based algorithm for both systolic and diastolic BP estimation (Table 4).

**TABLE 4:** Investigated machine learning approaches.

Approach	Algorithm
Regression	Linear regression
	Lasso regression
	Elastic net
Decision trees	Regression tree
	Forest regressor
k‐NN	k‐nearest‐neighbor regressor
Support vector machines	Support vector regressor
Ensemble learning	AdaBoost regressor
Deep learning	Multi‐layer perceptron

#### 4.4.2 Feature selection

In total, our dataset consisted of 50 unique features attributed to each cuff‐based BP measurement which we used to develop the estimation models. The ICG and time features were continuously recorded. To create the final dataset, to each of the reference (cuff‐based) BP values, the ICG values at the same time were asserted: (Supporting Information S2)

- Four biometric features (age, height, weight, and sex)
- Four BP features at calibration (calibration = first cuff‐based BP measurement at rest; systolic BP, diastolic BP, mean BP, and heart rate)
- 19 ICG features at the time of calibration
- 19 ICG features (as recorded in the instance)
- Four time features (sine/cosine transformed time of calibration + sine/cosine transformed time of measurement instance)

For each except from the deep learning model, we used a sequential feature selector algorithm to identify the best selection of features. To limit the need for computational resources, a maximum of 30 unique features was imposed on the search algorithm. The search optimized for a minimum RMSE (Supporting Information S3).^26^ For the deep learning algorithm, we tested the optimal features for each of the standard machine learning models and gradually adapted the selected features manually.

#### 4.4.3 Hyperparameter optimization

For all algorithms dependent on hyperparameter tuning, we used an extensive Grid Search algorithm to optimize the hyperparameters. Again, for computational reasons, we used a Hyperband search algorithm to perform hyperparameter tuning for the deep learning model.

#### 4.4.4 Evaluation of model performance

**FIGURE 8:**
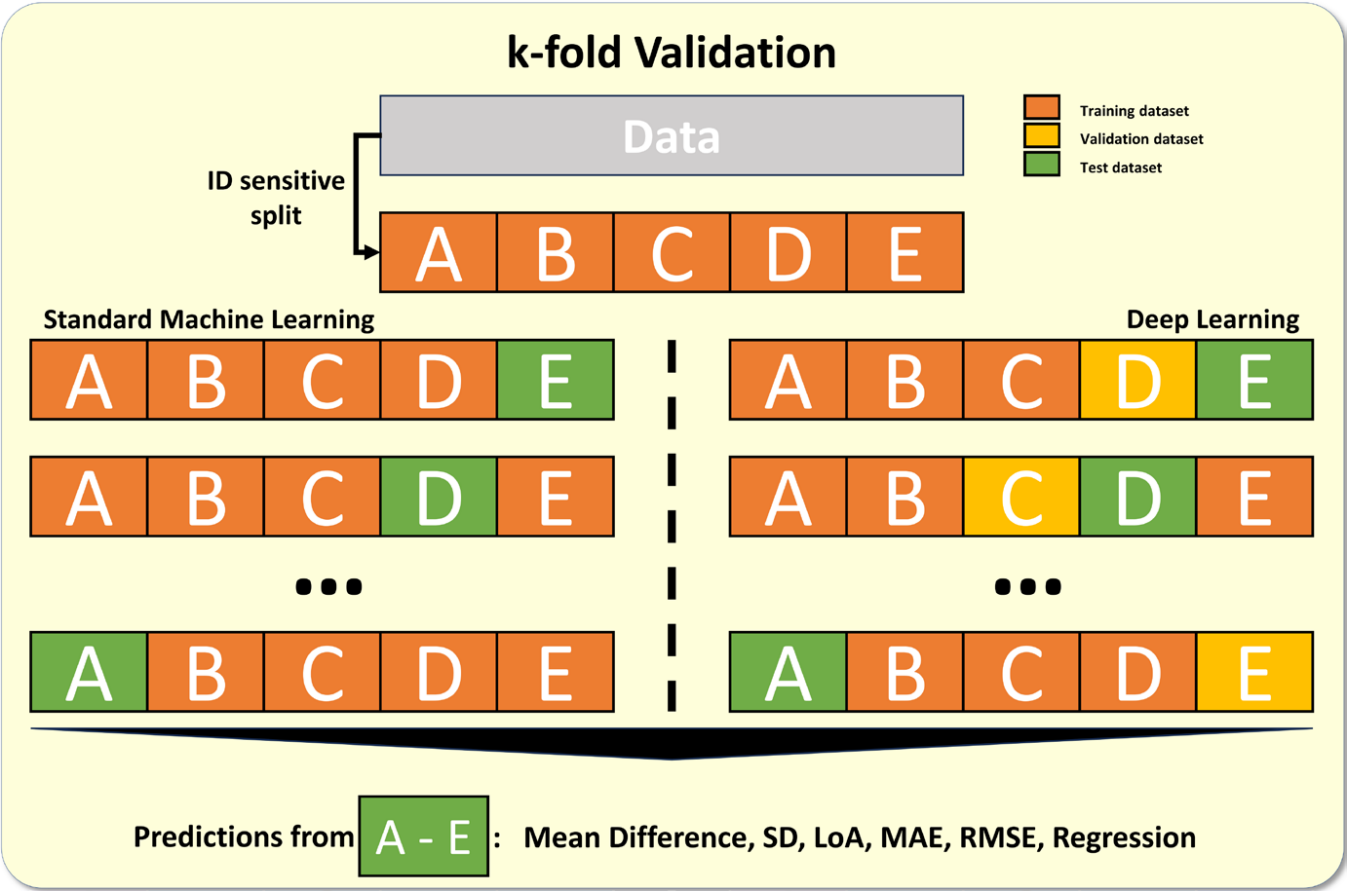
*k*‐fold Validation: The figure shows the *k*‐fold validation procedure used to evaluate the blood pressure estimation performance of the created models. The dataset was split into five different subsets (subject ID sensitive). Each model was trained five times on four of the five subsets and tested on the fifth. For the deep learning model, three subsets were used for model training, one for validation, and one for testing the model. The predictions (on the five test sets) were subsequently pooled and evaluation metrics were calculated. LoA, limits of agreement; MAE, mean absolute error; RMSE, root mean squared error; SD, standard deviation.

To evaluate the BP estimation performance, we used a *k*‐fold (fivefolds) validation scheme. Performance was evaluated by the *k*‐fold predictions and calculation of the RMSE. Subsequently, we calculated the total mean difference, standard deviation (SD), limits of agreement, and mean absolute error to improve the results' interpretability. By using the *k*‐fold validation procedure, we were able to derive reliable and comprehensive validation data (on the whole dataset) while rigorously maintaining a split of training and test data within each fold (Figure 8).

The results of all *k*‐fold validations were plotted in a linear regression analysis, to retrieve model specific correlation parameters and the best performing (lowest RMSE) models for systolic and diastolic BP were plotted in a Bland–Altman analysis, including a trend slope analysis to evaluate the data for possible bias trends.

In addition, we used the *B*‐score to evaluate the “true,” relative model performance. Designed to evaluate BP estimation performance, the *B*‐score compares the results obtained by a model to standardized performance estimates. These standardized estimates, called *base performances*, depict how easy it is to estimate BP from a dataset by analyzing its inter‐ and intraindividual BP variability. Any model scoring a defined *B*‐score (>0.0) provides measurement accuracy comparable to the accuracy provided by current cuff‐based ambulatory BP measurement devices.^5^, ^26^

We analyzed the best performing model's ability to track hypertension classifications by assessing its agreement with the cuff value derived classifications for rest (second rest) and for the average of all mental stress (TSST) measurements. Differences in derived mean BP values were analyzed using Bonferroni–Holm corrected paired *t*‐tests. The hypertension classifications are in accordance with the current ESH guidelines.^1^

The *B*‐score was calculated using the RMSE average from the *k*‐fold validation scheme. We also plotted a Bland–Altman analysis for the best performing model for both systolic and diastolic BP estimation.

#### 4.4.5 Analysis software

All analyses were conducted in Python 3, using the pandas,^27^ numpy,^28^ Scikit‐learn,^29^ SciPy,^30^ and TensorFlow^31^ libraries.

### 4.5 Ethics

The study was conducted with the approval of the local ethics committee (Ethics Committee of Charité—Universitätsmedizin Berlin, approval number EA4/051/21) and registered in the clinical trial register of the Charité—Universitätsmedizin Berlin (ePA: 3000224) and was performed in full accordance with the Helsinki Declaration.^19^

## 5 CONCLUSION

This study demonstrated the feasibility of using machine learning algorithms to estimate BP through ICG data. The tested models showed encouraging results in estimating both systolic and diastolic BP. However, standardized clinical validation and further development aimed at mitigating its limitations remains essential before this technology could be considered for clinical use. At present, diagnosing or monitoring arterial hypertension, or adjusting therapy based on ICG‐based BP estimation, is not recommended.

If validated and shown to be beneficial in larger clinical studies, ICG‐based BP estimation may hold potential as a clinical tool, offering a more comfortable alternative to conventional cuff‐based methods with more frequent BP measurements. Potential applications for an ICG‐based BP estimation system may lie in stationary settings with minimal patient movement, such as post‐surgical surveillance or the intensive care unit.

Future research should investigate more diverse patient cohorts in dynamic, real‐world settings. Additionally, observed biases should be addressed by employing more sophisticated models that rely on larger training datasets.

## AUTHOR CONTRIBUTIONS

**T. L. Bothe:** Conceptualization; investigation; writing – original draft; methodology; validation; visualization; writing – review and editing; software; formal analysis; project administration; data curation; supervision; resources. **A. Patzak:** Conceptualization; funding acquisition; writing – original draft; writing – review and editing; project administration; supervision. **O. S. Opatz:** Writing – review and editing; funding acquisition. **V. Heinz:** Investigation; writing – review and editing; formal analysis. **N. Pilz:** Formal analysis; writing – original draft; conceptualization; investigation; writing – review and editing; project administration; supervision; resources.

## FUNDING INFORMATION

This study was supported by the DLR (German Aerospace Center, Bonn, Germany) grant number 331 50WB2330 (T‐Mini+) and the BMWK (German Federal Ministry for Economic Affairs and Climate Action) grant number 16SV9251 (UniSensor).

## ACKNOWLEDGEMENT

There are no acknowledgements to be made for this article.

## CONFLICT OF INTEREST STATEMENT

T.L.B. and, A.P. advise SOMNOmedics on blood pressure.

## Supporting information


Appendix S1.

## Data Availability

The raw data supporting the conclusions of this article will be made available by the authors, without undue reservation.
