# Supplementary material for: Machine learning‐based blood pressure estimation using impedance cardiography data
Source: Acta Physiol (Oxf). 2025 Jan 13;241(2):e14269. doi: 10.1111/apha.14269 (PMC11726408; doi:10.1111/apha.14269)
Supplement: Supplementary file 1 — Appendix S1. [file APHA-241-e14269-s001.docx]

**Supplement Material**

**Supplement 1: Comprehensive description of impedance-cardiography measurement technology**

Impedance cardiography (ICG) is a non-invasive method for assessing cardiovascular function by measuring the electrical impedance of the thorax. The technique exploits the principle that blood, being a good conductor of electricity, alters the thoracic impedance as its volume changes during the cardiac cycle.

A small, high-frequency, low-amplitude electrical current is passed between subjects neck and their thoracic aperture. The electrodes measure the voltage drop across the thorax. By calculating the impedance, the technology can track changes in thoracic blood volume.

During each heartbeat, the heart ejects blood into the aorta, causing a decrease in thoracic impedance. This change in impedance is recorded as a waveform. Analyzing this waveform allows the determination of cardiovascular parameters. (see Supplement 2)

The process begins with the detection of the baseline thoracic impedance, primarily influenced by the tissue and fluid composition. As the heart pumps, the increased blood volume in the thoracic cavity decreases impedance, which is continuously monitored. The resulting data provides insights into the cardiac cycle, specifically the timing and volume of blood ejection.

**Supplement 2: Impedance-cardiography features available for BP models**

| **Parameter** | **Definition** | **Baseline Mean ± SD** |
| --- | --- | --- |
| **flow** |  |  |
| heart rate | heart beats per minute | 80.5 bpm ± 14.1 |
| heart period duration | time between two R-waves | 769.7 ms ± 130.6 |
| stroke volume | amount of blood ejected by the left ventricle of the heart in one heart beat | 81.8 ml ± 15.6 |
| stroke volume index | stroke volume relative to body surface area | 44.8 ml / m^2^ ± 6.4 |
| cardiac output | amount of ejected by the left ventricle within one minute | 6.5 l / min ± 1.3 |
| cardiac index | cardiac output relative to body surface area | 3.6 l / min / m^2^ ± 0.6 |
| **contractility** |  |  |
| velocity index | peak velocity of blood flow in the aorta during systole | 70272.0 / s± 15524.5 |
| acceleration index | maximum acceleration of blood flow in the aorta during systole | 13378.3 / s^2^ ± 3808.4 |
| Heather index | indicator for contractility | 18.9 ± 6.9 |
| pre ejection period | time delay between R-wave (ECG) and blood ejection into the aorta | 104.6 ms ± 13.0 |
| left ventricular ejection time | time interval from aortic valve opening to aortic valve closure | 262.2 ms ± 22.7 |
| systolic time ratio | pre ejection period divided by left ventricular ejection time | 0.40 ± 0.06 |
| systolic time ratio index | systolic time relative to body surface area | 0.54 / m^2^ ± 0.14 |
| ejection time ratio | left ventricular ejection time divided by heart period duration | 0.34 ± 0.05 |
| ejection time ratio index | ejection time ratio relative to body surface area | 3.91 / m^2^ ± 0.2 |
| O/C ratio | amplitude of the systolic wave divided by the amplitude of the diastolic wave | 34.7 ± 12.1 |
| **fluid** |  |  |
| thoracic fluid | indicator of thoracic fluid status | 29.3 / kΩ /m^2^ ± 3.8 |
| thoracic fluid index | thoracic fluid relative to body surface area | 16.2 / kΩ / m^2^ ± 1.9 |
| thoracic impedance | total thoracic impedance (alternating current resistance) of the thorax | 34.7 Ω ± 4.4 |

*Supplement 2: The table shows a description of the different 19 parameters obtained through impedance cardiography.*

**Supplement 3: Impedance-cardiography features used by best performing models for systolic BP**

| **Systolic BP (Linear Regression) features** | |
| --- | --- |
| demographic parameters | sex |
| ICG parameters at time of measurement | heart rate, stroke volume index,  cardiac output,  acceleration index,  pre-ejection period |
| ICG parameters at calibration | heart rate,  pre-ejection period |
| Cuff parameters at calibration | systolic BP,  mean arterial BP |

*Supplement 3: The table shows the different input features which were used for the pest performing model for systolic blood pressure. One demographic parameter, five ICG parameters at time of measurement, two ICG parameters at calibration and two cuff parameters were utilized. ICG = impedance-cardiography, BP = blood pressure*

**Supplement 4: Hypertension Classification Analysis**

**
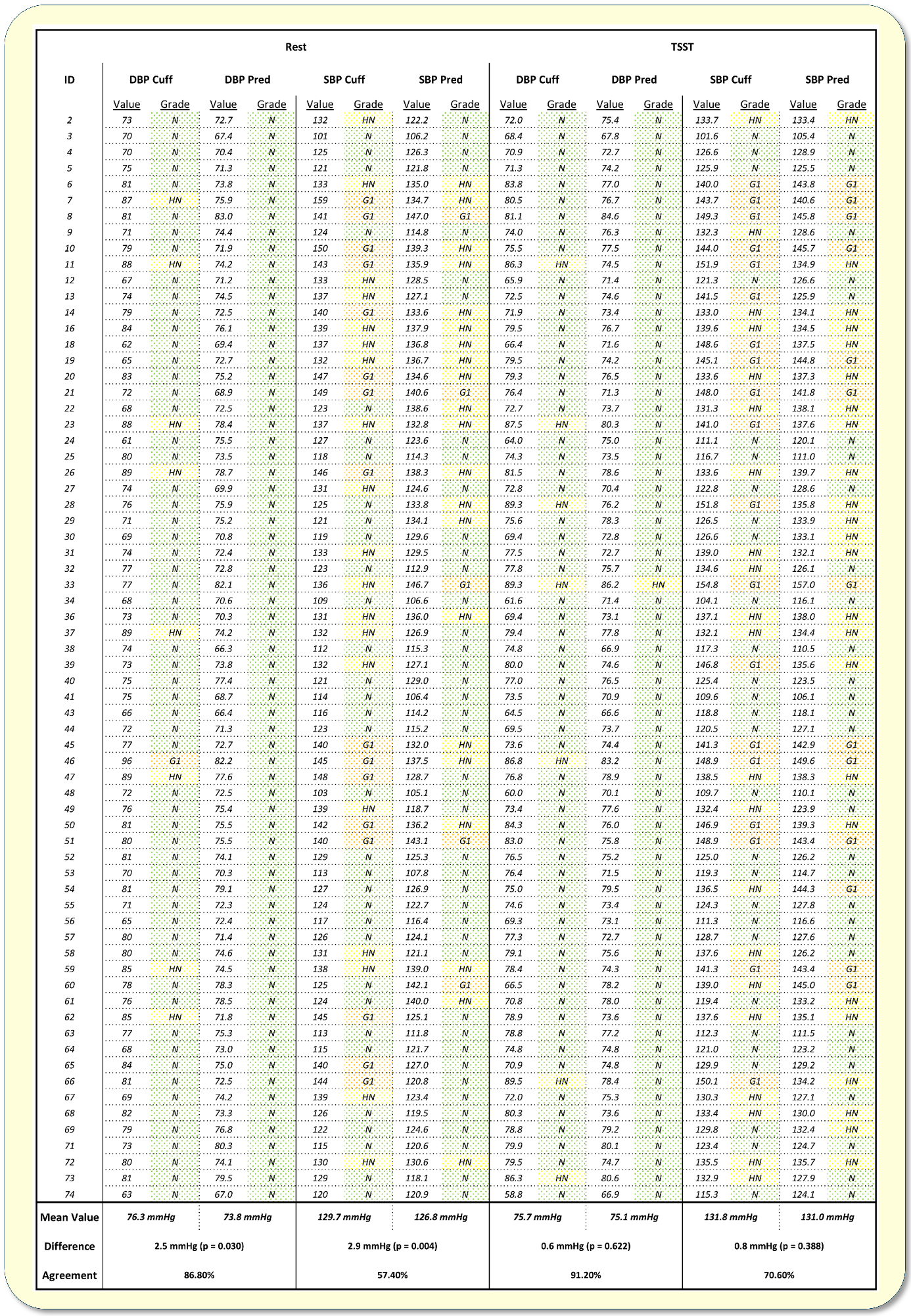
***Supplement 4: The figure shows the subject specific estimated and reference (cuff) blood pressure values for both systolic and diastolic blood pressure during the second rest phase (Rest, left) and during the mental load protocol (right, TSST). The derived hypertension grading is provided and highlighted in colour-coding. The mean of subject derived blood pressure values, the difference between the estimated and reference blood pressure (including p-values) and the classification agreement (Agreement) are provided at the bottom of the figure. TSST = Trier Social Stress Test, DBP = diastolic blood pressure, Pred = prediction (estimated blood pressure), SBP = systolic blood pressure, N = normal blood pressure, HN = high normal blood pressure, G1 = grade 1 hypertension.*

**Supplement 5: B-Score evaluation**
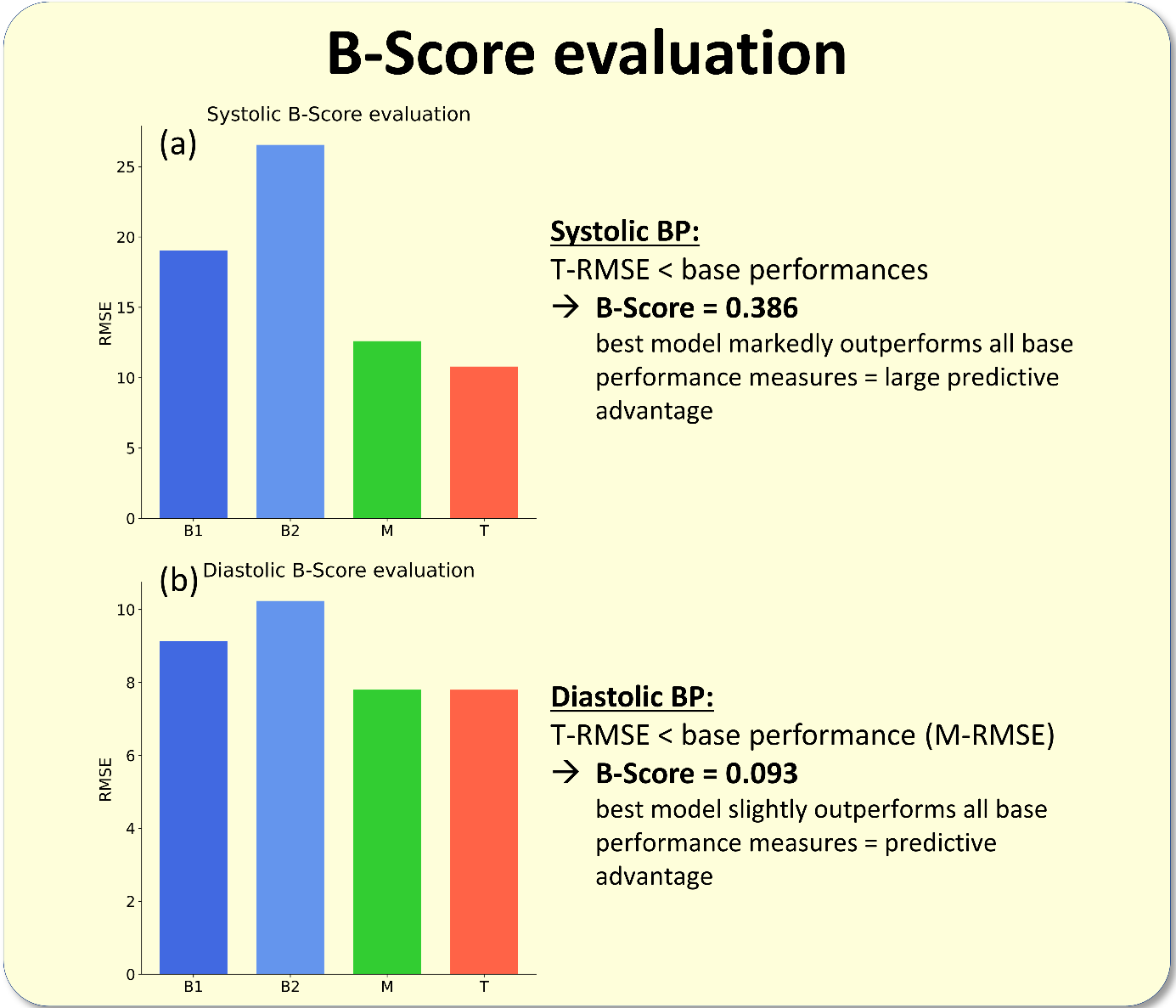


*Supplement 4: B-Score evaluation: The figure shows the base performances (B1-, B2-, and M-RMSE) and blood pressure estimation RMSE (T) for the systolic (Linear Regression, (a), upper panel) and diastolic (Support Vector Regressor, (b), lower panel) blood pressure. Both estimation models outperformed the base performances and therefore scored defined B-Scores > 0.0, indicating measurement accuracy comparable to validated, cuff-based devices used for ambulatory BP measurement.*
